# Proteomic Characterization of Murid Herpesvirus 4 Extracellular Virions

**DOI:** 10.1371/journal.pone.0083842

**Published:** 2013-12-30

**Authors:** Sarah Vidick, Baptiste Leroy, Leonor Palmeira, Bénédicte Machiels, Jan Mast, Sylvie François, Ruddy Wattiez, Alain Vanderplasschen, Laurent Gillet

**Affiliations:** 1 Department of Infectious Diseases, Faculty of Veterinary Medicine, University of Liège, Liège, Belgium; 2 Department of Proteomics and Microbiology, Research Institute for Biosciences Interdisciplinary Mass Spectrometry Center (CISMa), University of Mons, Mons, Belgium; 3 Electron Microscopy Unit, Veterinary and Agrochemical Research Centre, Brussels, Belgium; Ghent University, Belgium

## Abstract

Gammaherpesvirinae, such as the human Epstein-Barr virus (EBV) and the Kaposi’s sarcoma associated herpesvirus (KSHV) are highly prevalent pathogens that have been associated with several neoplastic diseases. As EBV and KSHV are host-range specific and replicate poorly *in vitro*, animal counterparts such as Murid herpesvirus-4 (MuHV-4) have been widely used as models. In this study, we used MuHV-4 in order to improve the knowledge about proteins that compose gammaherpesviruses virions. To this end, MuHV-4 extracellular virions were isolated and structural proteins were identified using liquid chromatography tandem mass spectrometry-based proteomic approaches. These analyses allowed the identification of 31 structural proteins encoded by the MuHV-4 genome which were classified as capsid (8), envelope (9), tegument (13) and unclassified (1) structural proteins. In addition, we estimated the relative abundance of the identified proteins in MuHV-4 virions by using exponentially modified protein abundance index analyses. In parallel, several host proteins were found in purified MuHV-4 virions including Annexin A2. Although Annexin A2 has previously been detected in different virions from various families, its role in the virion remains controversial. Interestingly, despite its relatively high abundance in virions, Annexin A2 was not essential for the growth of MuHV-4 *in vitro*. Altogether, these results extend previous work aimed at determining the composition of gammaherpesvirus virions and provide novel insights for understanding MuHV-4 biology.

## Introduction

The *Herpesviridae* is a large family of DNA viruses, including eight identified human herpesviruses. The *gammaherpevirinae* is one of the three subfamilies of the *Herpesviridae*. Two gammaherpesviruses are known to infect humans, Epstein-Barr virus (EBV) and the Kaposi’s sarcoma-associated herpesvirus (KSHV), which are associated with various types of cancer such as Burkitt’s lymphoma, nasopharyngeal carcinoma, Kaposi’s sarcoma or Castleman’s disease [1], [2]. Both EBV and KSHV have a very narrow host range *in vivo* and display limited lytic growth *in vitro*. These characteristics have hampered studies on these viruses and promoted the development of alternative models.

Murid Herpesvirus 4 (MuHV-4) is a relative of EBV and KSHV which has been isolated in wild rodents [3]. MuHV-4 offered therefore the possibility of developing a mouse model of gammaherpesvirus pathogenesis [3]–[5]. Moreover, in contrast to its human counterparts, MuHV-4 readily infects many types of cells *in vitro* and replicates to high levels. This virus has therefore been widely used as a model to study the gammaherpesviruses biology in host cells [6].

The MuHV-4 genome is estimated to encode at least 80 genes and is largely colinear with those of KSHV and EBV [7]. MuHV-4 virions display a morphological organisation which is typical of herpesviruses [8]. Briefly, infectious virions contain a double-stranded DNA genome which is incorporated in a large (diameter >100 nm) icosahedral nucleocapsid. This capsid is assembled in the nucleus from at least 8 different conserved proteins [9] and is surrounded by a thick proteinaceous tegument compartment which is acquired in both the nucleus and the cytoplasm. The tegument is probably the least understood part of herpesviruses although it plays important roles during entry, virion assembly and egress [10]–[12]. Recent studies have attempted to decipher its complex architecture [13]. The cytoplasmic capsids with tegument are finally enclosed within a lipid bilayer envelope spiked with glycoproteins to form mature infectious virus particles (diameter ∼200 nm). MuHV-4 encodes at least 9 envelope glycoproteins involved in virion entry and egress from the cell [14]–[21]. Among these glycoproteins, gB, gH, gL, gM and gN are shared by all the members of the *Herpesviridae* family [22].

In addition to virally encoded structural proteins, several cellular host proteins have also been reported in herpesvirus virions. While some of these proteins appear to be unique to a virus species, others are shared by several members of the *Herpesviridae* family. Although some cellular proteins incorporated in virions have been shown to be important for the biology of viruses from other families [23], the function of these proteins is still largely unknown in herpesviruses. A precise knowledge of herpesvirus composition is therefore likely to allow better understanding of various processes such as virus production, virion entry or immune evasion.

The usage of mass spectrometry-based analyses (MS) has allowed the deciphering of the composition of different herpesvirus virions [24]–[36]. Such an approach has been applied more than ten years ago to MuHV-4 virions [26]. However, this pioneering work revealed only 14 structural viral proteins whereas herpesvirus virions likely contain more than 30 different proteins [36]. Moreover, this study identified products of genes ORF20, ORF24 and ORF29 as virion proteins although their homologues were not found in most of the analyses of virions of related species. For example, ORF29 encodes a component of the viral terminase which is thought to dissociate from the capsid after genome packaging and therefore to not be incorporated in mature extracellular virions [31]. The composition of extracellular mature MuHV-4 virions deserves therefore new analyses.

In this study, 31 structural MuHV-4 proteins were identified by a recently described mass-spectrometry-based proteomic approach. In parallel, a small number of host proteins were also detected although their significance remains unclear. Altogether, these results extend our knowledge about gammaherpesvirus virion composition and provide novel insights for understanding MuHV-4 biology.

## Materials and Methods

### Cells and Virus

Baby Hamster Kidney cells (BHK-21 (ATCC CCL-10)) were cultured in Dulbecco’s modified Eagle’s medium (Invitrogen) containing 10% foetal calf serum (FCS), 2% Penicillin/Streptomycin (Invitrogen), 2 mM glutamine and 1% non-essential amino acids (Invitrogen). Mouse embryonic fibroblasts (MEF) from wild-type (WT) and Annexin A2-null mice [37] were kindly provided by Prof. Katherine A. Hajjar (Weill Cornell Medical College, New York, NY, United States) and were cultured in the same culture medium containing 1mM sodium pyruvate. Virions of the MHV-68 strain of MuHV-4 reconstituted from the pHA3 BAC plasmid [38] were used throughout this study. This viral strain therefore expresses eGFP under control of the immediate early promoter of human cytomegalovirus.

### Production and Purification of MuHV-4 Virions

BHK-21 cells were infected with MHV-68 at a multiplicity of infection (MOI) of 0.01 plaque-forming unit (PFU) per cell. To reduce cellular contaminants, the supernatant was harvested after 72 hours post-infection (hpi) before complete cell lysis. Extracellular virions were purified from the cell supernatant as described previously [36]. Briefly, after removal of the cell debris by low-speed centrifugation (1,000 *g*, 10 min at 4°C), virions present in the infected cell supernatant (∼1–5×10^6^ PFU/mL) were harvested by ultracentrifugation (100,000 *g*, 2 h at 4°C) through a 30% *weight* **/** *volume* (w/v) sucrose cushion. Virions were then banded by isopycnic gradient ultracentrifugation in a continuous 20 to 50% (w/v) potassium tartrate gradient in PBS (100,000 *g*, 2 h at 4°C). The band containing virions was collected (∼3 mL), diluted ten fold in PBS and pelleted by ultracentrifugation (100,000 *g*, 2 h at 4°C). The virion pellet was finally resuspended in PBS and virus enriched preparations (∼1–5×10^8^ PFU/mL) were stored at −80°C.

### Protease Treatment

Virions were treated with proteinase K as described previously [30]. Briefly, after ultracentrifugation through the sucrose cushion described above, the viral pellet was resuspended in 1 ml of MNT buffer (30 mM morpholineethanesulfonic acid [MES], 10 mM NaCl, and 20 mM Tris-HCl [pH 7.4]) containing 10 µg/ml proteinase K (Roche, Mannheim, Germany), incubated for 45 min at room temperature and subsequently treated with 2 mM phenylmethylsulfonyl fluoride (PMSF; Fluka) prior to density gradient centrifugation on a 20 to 50% (w/v) potassium tartrate gradient in PBS (100,000 *g*, 2 h at 4°C). The band containing virions was collected (∼3 mL), diluted ten fold in PBS and pelleted by ultracentrifugation (100,000 *g*, 2 h at 4°C). Proteinase K-treated virions were finally resuspended in PBS and stored at −80°C.

### Western Blotting

Purified virions were lysed and denatured by heating (95°C, 5 min) in Laemmli sample buffer (60 mM Tris-Cl pH 6.8, 2% (w/v) sodium dodecyl sulfate, 12.5% (w/v) glycerol, 5% (v/v) β-mercaptoethanol, 0.01% (w/v) bromophenol blue). Proteins were resolved by electrophoresis on Mini-PROTEAN TGX (Tris-Glycine eXtended) precast 4–15% resolving gels (Bio-Rad) in an SDS-PAGE running buffer (25mM Tris-base, 192 mM glycine, 0.1% (w/v) SDS) and transferred to polyvinylidene difluoride membranes (Immobilon-P transfer membrane, 0.45 µM pore size, Millipore). The membranes were blocked with 3% (w/v) non-fat milk in PBS/0.1% (w/v) Tween-20, and then incubated in the same buffer with rabbit polyserum [5] or mouse monoclonal antibodies (mAb) recognizing MuHV-4. We used the following mAbs: 3F7, anti-gN IgG_2a_ [39]; T1A1, anti-gp150 IgG_2a_ [40]; 58-16D2, anti-gp70 IgG_2a_ [15]; MG-4D11, anti-gB IgG_2a_; MG-2C10, anti-gB IgM [41]. Bound antibodies were detected with horseradish peroxidase-conjugated goat anti-rabbit IgG pAb or anti-mouse IgG pAb (Dako Corporation), followed by washing in PBS/0.1% Tween-20, development with ECL substrate (GEHealthcare) and exposure to X-ray film.

### Fractionation of MuHV-4 Virions

Lipid envelopes were removed from capsids-teguments by incubation with a non-ionic detergent as described previously [42]. Briefly, virions enriched preparations were sonicated and lysed in PBS containing 1% (v/v) NP-40 for 20 min on ice. Capsids associated with tegument were then pelleted by centrifugation (15,000 *g*, 30 min at 4°C). The supernatant was discarded and the procedure was repeated two times on the capsid-tegument pellet. After the last centrifugation, the capsid-tegument pellet was finally resuspended in PBS and stored at −80°C until further use.

### Negative Staining and Electron Microscopy

Copper grids (400 mesh; Agar Scientific) covered by a thin film of pioloform were incubated for 10 min with 1 % Alcian blue 8G solution (Gurr Microscopy Materials, BHD) to add positive charges. After washing, virion-enriched or capsid-tegument preparations were adsorbed to the grids for 10 min. Viral particles were then stained by incubation on a 2 % uranyl acetate solution for 10 s (Agar Scientific). Samples were observed using a transmission electron microscope (FEI Tecnai Spirit).

### Oligosaccharide Digestion

The deglycosylated extract was obtained by treating virion-enriched preparations with an Enzymatic Protein Deglycosylation kit (Sigma) following the instructions of the manufacturer. Viral proteins were successively denatured for 5 min at 100°C in a denaturation solution, and treated with Triton X-100, peptide : *N*-glycosidase *F*, *O*-glycosidase, *α*(2–3,6,8,9)neuraminidase, *β*(1,4)-galactosidase and *β*-*N*-acetylglucosaminidase for 3 h at 37 °C. This extract was then submitted to the one dimensional gel electrophoresis/Nanoscale liquid chromatography coupled to tandem mass spectrometry approach (1D gel/nanoLC-MS/MS approach).

### 1D gel/nanoLC-MS/MS Approach

Proteins from virion-enriched (treated or not with proteinase K, deglycosylated or not) and from capsid-tegument preparations were extracted in Laemmli sample buffer and heated for 5 min at 95°C. The samples were then separated by SDS-PAGE on 4–20% acrylamide 7 cm gels (Invitrogen). Separated proteins in the gel were excised in 30 serial slices along the lane. Gel slices were submitted to in-gel digestion with sequencing grade modified trypsin as described previously [33], [36]. Briefly, gels were washed successively with 50 mM ammonium bicarbonate (ABC) buffer and ABC buffer/acetonitrile (ACN) 50% (v/v). Proteins were reduced and alkylated using dithiothreitol and iodoacetamide followed by washing with ABC and ABC/ACN. Resulting peptides were analysed by nanoLC-MS/MS using a 40 min ACN gradient as described [43].

### MS/MS Analyses

Peptides were analysed using the ‘peptide scan’ option of the HCT Ultra ion trap (Bruker), consisting of a full-scan MS and MS/MS scan spectrum acquisitions in ultrascan mode (26 000 *m*/*z* s^−1^). Peptide fragment mass spectra were acquired in data-dependent AutoMS(2) mode with a scan range of 100–2800 *m*/*z*, three means and four precursor ions selected from the MS scan 300–1500 *m*/*z*. Precursors were actively excluded within a 0.5 min window, and all singly charged ions were excluded. Peptide peaks were detected and deconvoluted automatically using Mascot distiller 2.3.3 and submitted to database search using an in-house mascot search engine (2.2 version). The default search parameters used were the following: Enzyme = Trypsin; Maximum missed cleavages  = 2; Fixed modifications = Carbamidomethyl (C); Variable modifications = Oxidation (M); Peptide tolerance ±1.5 Dalton (Da); MS/MS tolerance ±0.5 Da; Peptide charge  = 2+ and 3+; Instrument = ESI-TRAP. All data were searched against the NCBI Rodents database in order to detect host proteins or against a MuHV-4 database [44] to detect viral proteins. Proteins were only considered if reaching a p-value for identification lower than 0.05. Only peptides with a mascot score higher than 30 were taken into account. Identifications based on single peptide were validated manually considering that a continuous series of at least 5 b or y ions had to be present and that selected ions had to be among the top 15 ions of the spectrum. For each approach, the exponentially modified protein abundance index (emPAI) [45] was calculated to estimate protein relative abundance for the complete virion extracts. The protein abundance index (PAI) is defined as the number of observed peptides divided by the number of observable peptides per protein. The exponentially modified PAI (10^PAI^ - 1) is proportional to protein content in a protein mixture in LC-MS/MS experiments.

### Proteogenomic Mapping

The complete nucleotide sequence of MuHV-4 (GenBank: AF105037.1) was translated *in silico* in all 6 frames. The mass lists obtained in the different complete virion analyses were searched against these 6 frames with no mismatches allowed. Detected peptides were then mapped onto the original MuHV-4 genome sequence (GenBank: AF105037.1 and [7]). The results were generated and visualized using R [46] and the seqinr package [47].

### Growth Curves

Cell cultures were infected with MuHV-4 virions at a MOI of 0.01 (multi-step assay). After 1 h of adsorption, the cells were washed then overlaid with Dulbecco’s modified Eagle’s medium containing 5% FCS. Supernatants of infected cultures or infected cells were harvested at successive intervals and the amount of infectious virus determined by plaque assay on BHK-21 cells.

## Results

### Purification of Extracellular MuHV-4 Virions

**Figure 1:**
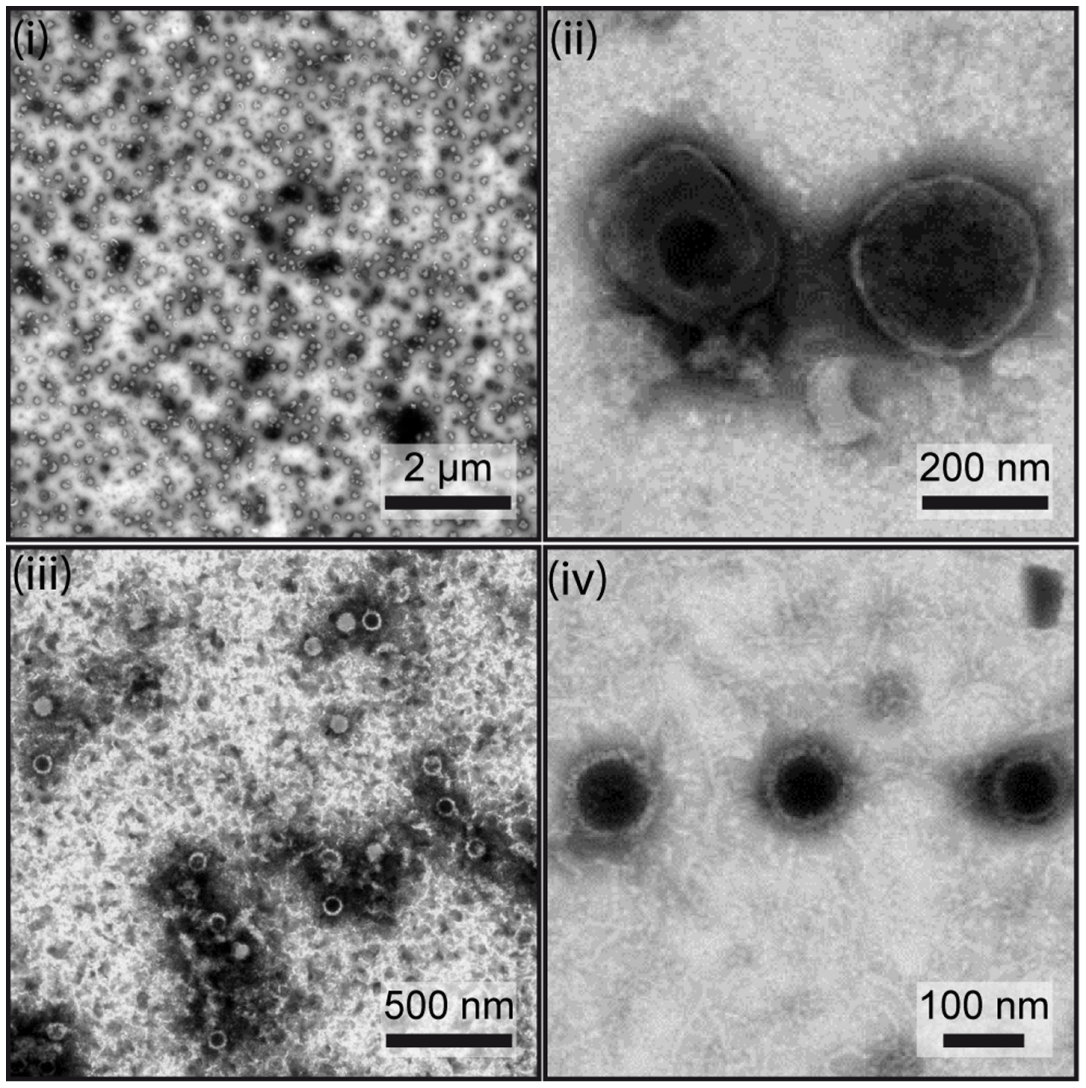
Assessment of MuHV-4 virion purity. The purity of the virions (i and ii) and capsid-tegument fractions (iii and iv) preparations was assessed by negative staining and electron microscopy. Scale bars are shown on the images.

The purification of extracellular mature virions is very important for the characterization of viral structural proteomes. Indeed, it has to be very pure to avoid detection of non-structural proteins but in the same time to be highly concentrated. In the present study, we used a strategy recently used for the characterization of BoHV-4 virions [36]. Briefly, MuHV-4 extracellular virions were harvested and purified from the supernatant of BHK-21 infected cells before complete lysis as described in the Materials and Methods. Transmission electron microscopic (TEM) analysis of negatively stained samples was used to evaluate both the concentration and the purity of all the preparations (Fig. 1). In all samples, typical herpesvirus virions were observed. These appear as spherical to slightly pleomorphic, relatively electron-lucent features of 120–200 nm in diameter. These virions with an intact envelope displayed no specific internal and surface features in negative staining. Because of the drying during the negative staining, some of the virions collapsed such that the envelope was penetrated by the stain (Fig. 1, panel ii). In that case, the nucleocapsids were observable, surrounded by the integument and the envelope. Only a few nucleocapsids without envelope were found (1 to 5% in the different samples). Most likely, these had no envelope or lost their envelope during virus purification. We did not observe any contamination by cell debris, suggesting that the virion samples were very pure. This therefore indicates that our virion purification strategy could be considered as successful, at least as evaluated by TEM.

### Viral Protein Composition of MuHV-4 Virions

In order to analyse MuHV-4 virion composition, virion proteins were separated by SDS-PAGE, digested in gel with trypsin and analysed by mass spectrometry (nanoLC-MS/MS). This approach allows association of the identified protein with the apparent molecular mass assessed by SDS-PAGE. This approach was performed on untreated virions or on intact virions treated with proteinase K (proteinase K - 1D gel/nanoLC-MS/MS). We also applied the same protocol on deglycosylated virion extracts.

This approach enabled us to identify 31 virally encoded proteins in the MuHV-4 particle. These proteins are listed in Table 1 according to their position in the viral genome. This number is consistent with the numbers previously reported for other members of the *Herpesviridae* family. Moreover, it substantially improved a previous analysis of MuHV-4 virions that identified 14 virally encoded structural proteins [26].

**Table 1:** Viral content of MuHV-4 extracellular virions.

				1D nanoLC-MS/MS^a^				Deglyco-1D nanoLC-MS/MS			
ORF	Protein description	Predicted MM (kDa)	pK^b^	No. of peptides^c^	Prot matches^d^	Coverage (%)^e^	emPAI (% of max)^fg^	No. of peptides^c^	Prot matches^d^	Coverage (%)^e^	emPAI (% of max)^fg^
M3	Chemokine binding protein	44.94	+	11	27	36.45	19				
ORF4	Glycoprotein gp70	42.98	+	9	72	22.68	28	1	3	2,58	2
**ORF8** h	**Glycoprotein B**	96.53	+	33	121	53.00	28	29	141	49,12	58
ORF11	Potential tegument protein	43.07	+	12	64	53.09	27	4	10	20,10	9
ORF17	Minor scaffold protein (protease)	59.37	+	3	5	5.33	2				
ORF19	Capsid vertex-specific complex protein	57.71	+	8	23	20.93	9	4	9	12,79	6
ORF21	Thymidine kinase. potential tegument protein	72.85	+	39	471	68.01	80	14	65	29,04	25
**ORF22**	**Glycoprotein H**	83.75	+	29	173	40.96	46	15	82	31,10	22
ORF23	Egress protein	42.97	+	18	99	72.97	68	10	22	46,19	34
**ORF25**	**Major capsid protein**	154.38	+	76	526	71.30	**100**	49	554	51,27	**100**
**ORF26**	**Triplex component**	33.84	+	13	99	61.20	127	11	58	60,87	142
ORF27	Glycoprotein gp48	29.65	+	9	86	36.61	43	5	10	24,80	23
**ORF28**	**Potential glycoprotein**	8.59	+	1	17	18.67	19	1	3	18,67	21
ORF32	Capsid vertex-specific complex protein	50.64	+	2	17	8.78	2	2	2	6,98	3
ORF33	Teg. myristoylated protein binding protein	36.70	+	13	205	46.48	90	10	83	44,34	58
ORF36	Kinase	49.93	+	3	5	9.61	2	1	1	2,97	1
ORF38	Tegument myristoylated protein	8.34	+	1	4	10.67	7	1	2	10,67	9
ORF39	Glycoprotein M	43.91	+	9	137	19.84	36	7	60	18,80	19
ORF43	Capsid portal protein	64.61	+	2	2	4.23	1	1	1	4,06	1
**ORF45**	**IRF-7 binding prot. pot. tegument protein**	22.51	+	3	8	42.23	6				
ORF47	Glycoprotein L	15.35	+	4	15	48.18	14	2	10	24,82	26
ORF51	Glycoprotein gp150	50.03	+	1	30	5.18	3	1	3	5,18	1
**ORF52**	**Tegument protein**	14.85	+	12	417	64.44	5298	8	133	60,74	2312
ORF55	Tegument palmitoylated protein	21.01	–	2	4	25.79	4				
ORF58	Glycoprotein	40.62	+	2	2	6.05	1	1	1	3,17	2
ORF62	Triplex component	36.98	+	27	137	88.99	177	16	60	66,97	116
ORF63	Large tegument protein binding protein	103.31	+	2	3	2.66	1	1	1	1,77	1
ORF64	Tegument protein	274.62	+	15	32	12.94	2	6	20	3,62	2
ORF65	Small capsomer interacting protein	19.99	+	15	121	85.56	503	8	17	52,69	67
**ORF75C**	**Tegument protein/v-FGAM-synthetase**	147.26	+	52	656	54.35	46	36	478	39,47	52
ORF75B	Tegument protein/v-FGAM-synthetase	144.37	+	36	390	46.27	25	25	202	30,12	30

^a^ This approach was accomplished in three independent biological replicates and results were pooled.

^b^ Proteinase K treatment. +. proteins detected in the Proteinase K - 1D gel/nanoLC-MS/MS.

^c^ Number of unique peptides identified per protein.

^d^ Number of peptides detected per protein.

^e^ Values shown are the percentages of coverage of proteins by peptides.

^f^ emPAI values were calculated as described by Ishihama *et al.* [42].

^g^ Relative emPAI values were calculated as percentages of pORF25 abundance for each individual replicate and mean values were calculated.

^h^ Proteins previously identified in MuHV-4 virions [26] are highlighted in bold.

**Figure 2:**
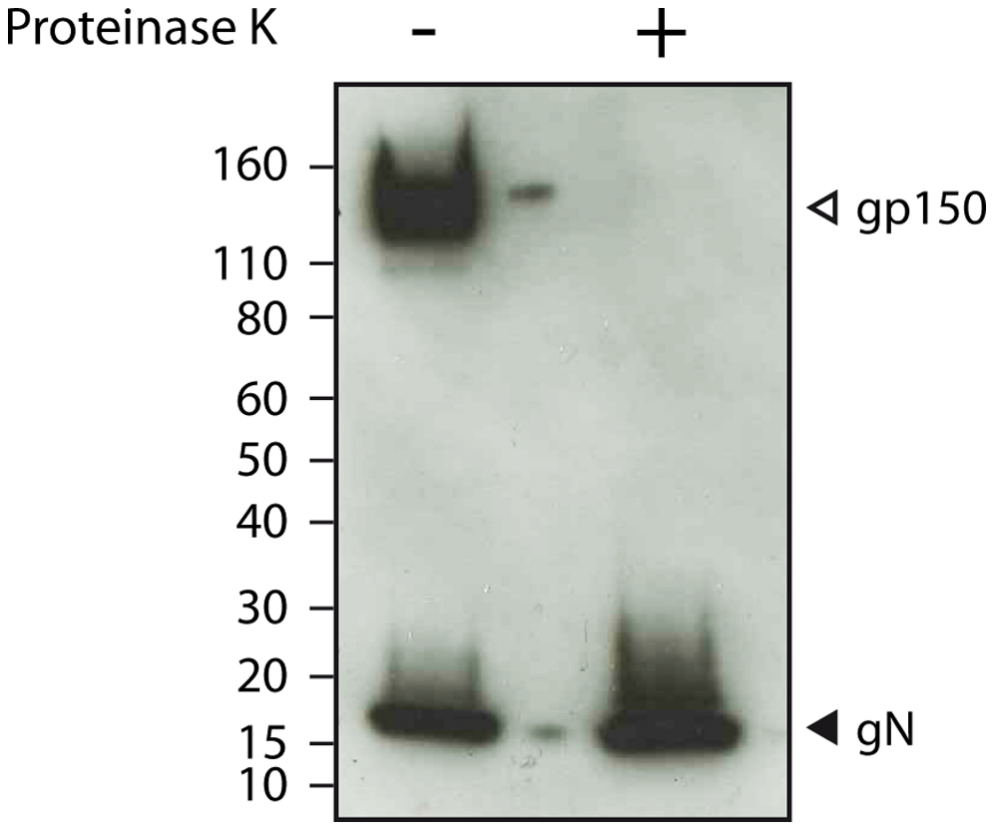
Sensitivity of viral proteins to proteinase K treatment of virions. Western blotting analysis of viral proteins in purified virions from mock (–) and proteinase K (+) treated samples. Gp150 and gN are type I transmembrane proteins which have a N-terminal domain predicted to be sensitive to proteinase K digestion.

Despite our multistep purification protocol, detection of some proteins could result from non-specific sticking to the virion rather than true integration into the particle. To address this issue, we treated virions with proteinase K, in the absence of detergent, prior to density centrifugation as described previously [30], [32], [36]. We validated this treatment by western blotting (Fig. 2). As herpesviruses are enveloped within a phospholipid bilayer, only proteins that are exposed at the viral surface are accessible to protease digestion. Immunoblotting with MuHV-4 specific monoclonal antibodies confirmed that some epitopes disappeared after proteinase K treatment while some others were not affected (Fig. 2). Thus, the epitope recognized by mAb T1A1 (recognizing gp150) was detected only in untreated virions while it was undetectable in proteinase-K treated virions (Fig. 2). This therefore suggests that our proteinase K treatment was efficacious. In contrast, the viral surface epitope recognized by mAb 3F7 raised against gN, which is a small (61 aa among which only 29 aa are exposed at the cell surface) type I membrane protein lying probably very close to the envelope, did not appear to be affected by the treatment (Fig. 2).

Among the 31 proteins described in Table 1, only pORF55 was not detected in the proteinase K - treated sample. Analysis of the peptide sequence coverage of viral proteins identified by mass spectrometry revealed that, although some MuHV-4 envelope glycoprotein epitopes were sensitive to proteinase K digestion (Fig. 2), proteinase K treatment did not affect the detection of most of the viral proteins even those that are exposed at the surface of the virion. Thus, the peptide sequence coverage of gB, which is the envelope protein detected by the bigger number of unique peptides (Table 1), was 51.8% and 53% respectively in untreated and proteinase K-treated virions. Finally, no additional protein was specifically detected after proteinase K treatment.

### Estimation of Protein Abundance in MuHV-4 Virion

We previously used the emPAI value (exponentially modified protein abundance index) developed by Ishihama *et al.* to estimate the protein contents in our samples [36], [45], [48]. In order to relatively quantify viral proteins in virions, emPAI values were expressed as percentages of the emPAI value obtained for pORF25, the major capsid protein (Table 1). Based on these relative emPAI values, the tegument protein encoded by ORF52 was, by far, the most abundant protein detected in MuHV-4 virions. It was more than 20 times more abundant than pORF25. This apparent abundance could not be biased through the enhanced detection of a single peptide as 12 different pORF52 peptides were detected in similar proportions by our analyses (Table 1).

### Identification of Viral Capsid and Capsid Associated Tegument Components

In order to identify capsid proteins and tegument proteins associated with capsids, we purified viral capsid-tegument fractions as described in the Materials and Methods. We estimated the purity and concentration of the purified capsid-tegument preparation by TEM (Fig. 1, panels iii and iv). As expected, the sample contained only isolated capsids and no trace of intact virions or envelope debris.

The 1D gel/nanoLC-MS/MS approach revealed the presence of 18 viral proteins in this sample (Table 2). As expected, no predicted viral envelope glycoprotein (encoded by ORF4, ORF8, ORF22, ORF27, ORF28, ORF39, ORF47, ORF51 and ORF58) was detected in this sample confirming the quality of our purification procedure. Moreover, proteins encoded by M3, ORF38, ORF45 and ORF55 were also not detected in this sample although they had been detected in complete virions. For ORF38, ORF45 and ORF55, the absence of detection could be associated with their low abundance as very few peptides derived from these proteins were detected in intact virions. The absence of detection could also reflect the fact that they are not directly associated with capsids. In this case they could be considered as outer tegument proteins. Regarding M3, as it is a secreted protein, its absence from tegument was expected.

**Table 2:** Proteins of MuHV-4 virions identified by 1D gel/nanoLC-MS/MS as associated with purified capsid-tegument samples.

ORF	Protein description	Predicted MM (kDa)	Proteinase K^a^	No. of peptides^b^	Prot matches^c^	Covergae^d^	emPAI (% of max)^f^	Expected relative abundance in capsid (%)^g^
ORF11	Potential tegument protein	43.07	+	11	31	52,32	14,04	
**ORF17** h	**Minor scaffold protein (protease)**	59.37	+	7	35	14,34	3,16	15.7
**ORF19**	**Capsid vertex-specific complex protein**	57.71	+	10	28	31,20	7,02	up to 6.28
ORF21	Thimidine kinase	72.85	+	23	200	50,31	24,97	
ORF23	Egress protein	42.97	+	14	44	51,71	24,02	
**ORF25**	**Major capsid protein**	154.38	+	70	527	70,65	100,00	100
**ORF26**	**Capsid triplex component**	33.84	+	11	73	60,87	91,32	67
**ORF32**	**Viral DNA cleavage/packaging protein (Capsid vertex-specific complex)**	50.64	+	6	19	20,72	5,97	up to 6.28
ORF33	Tegument protein	36.70	+	9	45	44,04	12,79	
ORF36	Kinase	49.93	+	6	11	17,16	3,31	
**ORF43**	**Portal protein**	64.61	+	20	29	47,97	16,45	1.25
ORF52	Tegument protein	14.85	+	10	129	69,63	1066,10	
**ORF62**	**Capsid triplex component**	36.98	+	20	96	86,54	175,63	33.5
ORF63	Tegument protein	103.31	+	14	33	21,82	3,46	
ORF64	Tegument protein	274.62	+	41	78	25,56	3,96	
**ORF65**	**Small capsomer interacting protein**	20.00	+	11	77	75,81	282,00	94.2^i^
ORF75C	Tegument protein/v-FGAM-synthetase	147.26	+	39	287	45,95	14,99	
ORF75B	Tegument protein/v-FGAM-synthetase	144.37	+	32	147	41,33	9,43	

^a^ Proteinase K treatment. +, proteins detected in the Proteinase K - 1D gel/nanoLC-MS/MS analysis of virions.

^b^ Number of unique peptides identified per protein.

^c^ Number of peptides detected per protein.

^d^ Values shown are the percentages of coverage of proteins by peptides.

^e^ emPAI values were calculated as described by Ishihama *et al.*, [42].

^f^ Relative emPAI values were calculated as percentages of pORF25 abundance.

^g^ As described for HSV-1B- capsids by Baines *et al*., [9].

^h^ Predicted capsid proteins are highlighted in bold.

^i^ On the basis of full occupancy, i.e. one copy decorating each of 6 hexon tips.

As performed above for entire virions, the relative protein abundances were estimated by emPAI values. These values were expressed as percentages of the emPAI value obtained for pORF25, the major capsid protein (Table 2). As observed for intact virions, the tegument protein encoded by ORF52 was by far the most abundant protein detected in this preparation.

### Proteogenomic Mapping

**Figure 3:**
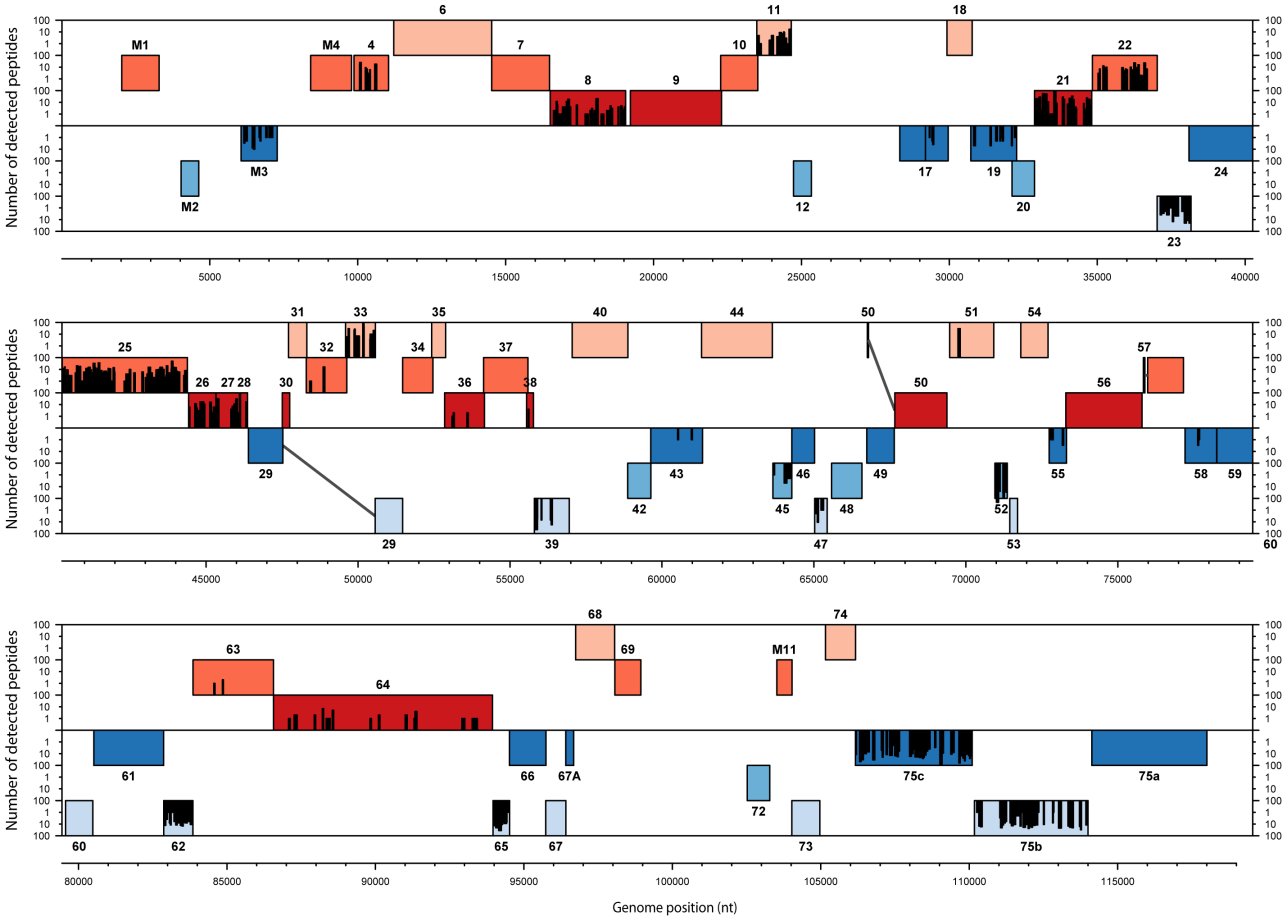
Proteogenomic map of MuHV-4 structural peptides. The 6 possible frames of the MuHV-4 genome (AF105037.1) translation are shown with rectangle indicating annotated ORFs. Red and blue ORFs represent forward and reverse frames respectively. Detected peptides are shown by bars whose the height is proportional to the number of detections. Genomic positions in bp are indicated.

Our identification of MuHV-4 structural proteins by mass spectrometry is based on the ORFs annotations of the MuHV-4 genome. In order to identify possible MuHV-4 virions proteins that had not been annotated, we generated a database containing the entire genome of MuHV-4 translated in the 6 frames. The mass lists obtained in the different complete virions analyses were searched against this database as described in the Materials and Methods. Finally, the detected peptides were graphically mapped onto the MuHV-4 genome (Fig. 3). The results obtained showed that all peptides identified by our analyses mapped into previously annotated ORFs.

This approach allowed us to easily analyse the localization of detected peptides within protein sequences. This was particularly useful for the ORF17–17.5 proteins (Fig. 3 and S1). Rhadinoviruses ORF17.5 encode the homolog of HSV-1 VP22a which serves as a scaffold protein during capsid maturation and is therefore mainly found in procapsids and B capsids [9]. As observed in other herpesviruses, the coding sequence of this protein is entirely contained within and in frame with a larger open reading frame, called ORF17 in rhadinoviruses. This larger open reading frame encodes a protease (homologous to HSV-1 VP24) involved in capsid maturation by degrading scaffold proteins. Interestingly, analysis of the ORF17–17.5 peptides distribution showed that these peptides were located into the ORF17 specific N-terminal region. These results suggest therefore that the pORF17 protease was the major ORF17-17.5 product present in our virion preparation. However, it has to be noted that some ORF17.5 peptides were found in the capsid-tegument preparation, some trace of the scaffold protein were therefore detected.

### Glycosylation of Virion Components

In order to improve the sensitivity of our approach, we performed the 1D gel/nanoLC-MS/MS analysis on deglycosylated virion proteins as described in the Materials and Methods. We validated this treatment by western blotting (Fig. S2). The apparent molecular mass of glycoprotein gp70 and of the C- and N-terminal parts of gB appeared to be reduced while the total protein content revealed by an anti-MuHV-4 polyserum did not appear to be affected (Fig. S2). However, compared to the analysis of intact virions, we were not able to detect any new viral structural protein (Table 1). In contrast, peptides corresponding to proteins encoded by M3, ORF17, ORF45 and ORF55 were not detected in the deglycosylated sample.

The comparison of results obtained by the 1D gel/nanoLC-MS/MS approaches performed on untreated and deglycosylated samples run in parallel allowed us to predict glycosylation of several virion proteins. We limited our analysis on detected envelope proteins as most of them are predicted to be N- and/or O-glycosylated (Fig. S3). Briefly, both samples were submitted to 1D gel electrophoresis as described in the Materials and Methods. After protein migration, the gels were cut in 30 slices and protein composition of each of the 30 slices was determined as described. Distribution across the gel was then determined for each protein and compared between deglycosylated or untreated sample.

**Figure 4:**
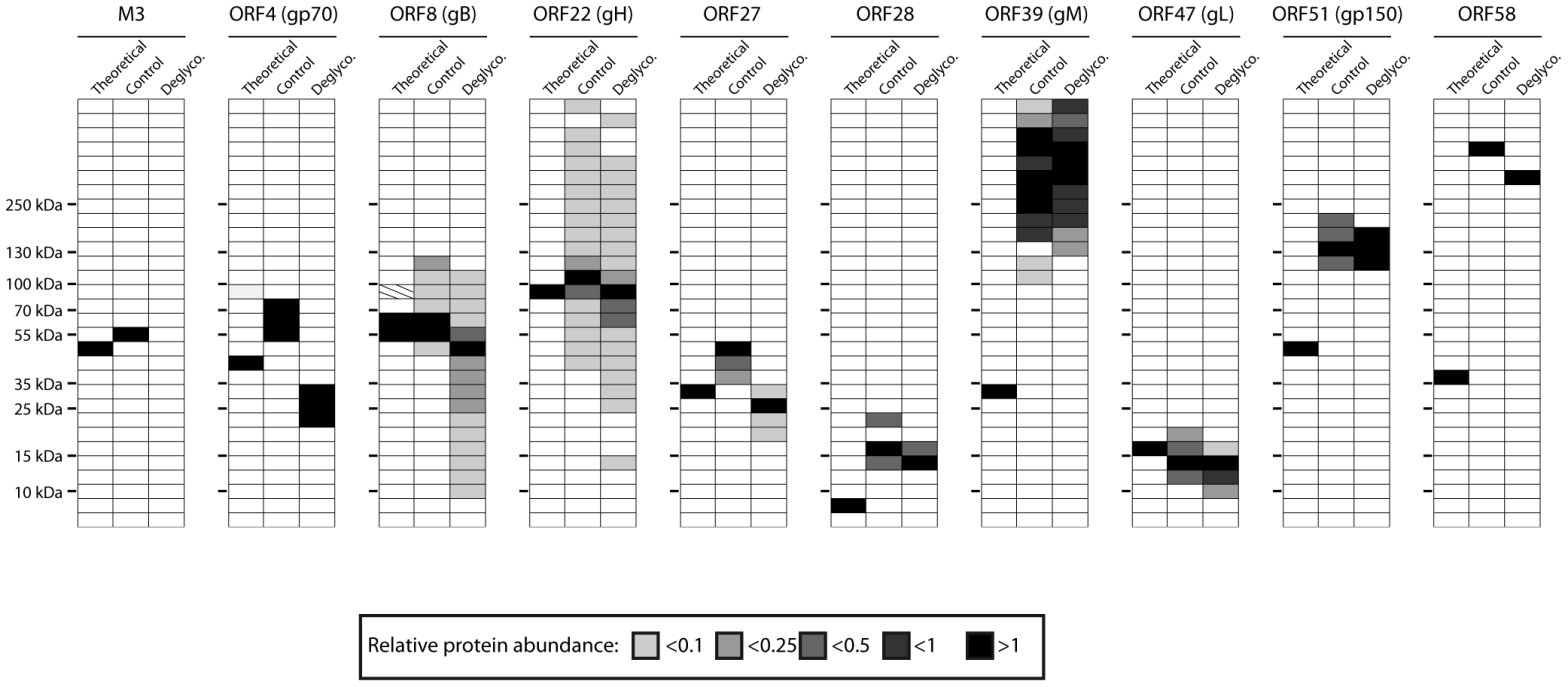
Analysis of MuHV-4 structural protein glycosylation. Control or deglycosylated proteins of purified MuHV-4 virions were separated by SDS-PAGE. After migration, each sample was divided in 30 serial slices along the lane and proteins of each slice were identified as described in the Materials and Methods. For the two treatments, slices containing M3, ORF4 (gp70), ORF8 (gB), ORF22 (gH), ORF27 (gp48), ORF28, ORF39 (gM), ORF47 (gL), ORF51 (gp150) and ORF58 proteins are shown with color intensity indicating the mean relative abundance (emPAI). For each lane, the abundances are expressed relatively to the band displaying the highest emPAI which is taken as 1. For each protein, predicted molecular mass is shown (theoretical lane). For the gB protein, the dashed slice indicated the predicted position of gB proteins before potential cleavage by cellular furin. The position of a MM standard is shown on the left.

In comparison with their theoretical mass (calculated from amino acid composition), all the proteins, excepted gL, displayed a higher apparent molecular mass, suggesting important posttranslational modifications (Fig. 4). We considered that there were differences of glycosylation when detection profiles differed by more than one positive box. In the case of the proteins encoded by ORF4 (gp70), ORF8 (gB), ORF22 (gH), ORF27 (gp48), ORF47 (gL) and ORF58, the deglycosylation procedure induced a decrease of the apparent molecular mass, suggesting that these proteins are glycosylated. It should be noted that MuHV-4 gB has a theoretical molecular mass (MM) of ∼96 kDa. However, MuHV-4 gB has a furin consensus cleavage sequence at amino acids 424 to 427 (RRKR) [49]. It has been shown experimentally that the cleavage is indispensable for virion incorporation and results in two derived products of respectively 55 and 65 kDa [49].

Although N- and/or O-glycosylation had previously been shown for pORF28 and pORF51 (gp150), our experimental procedure did not allow us to detect it. In the case of pORF51 (gp150), this is probably associated with an aberrant migration of the protein. This could be due to the high proline content (23%) of the protein, which is known to increase the apparent molecular mass on SDS-PAGE gels [50]. pORF39 (gM) and pORF58 also migrate to aberrant positions. This could be associated with the multiple transmembrane domains of these proteins [14], [39]. This could also reflect the formation of protein aggregates due to the conditions used in SDS-PAGE preparation as it has previously been shown for KSHV gM [51].

Although peptides corresponding to pM3 had been observed in all the replicate analyses that were performed on intact virions, no M3-derived peptides were detected in the deglycosylated sample (Table 1 and Fig. 4).

### Host Proteins Associated with MuHV-4 Extracellular Virions

Several host proteins have been detected in mature herpesvirus virions. We therefore also searched for proteins of non-viral origin in the 1D gel/nanoLC-MS/MS analyses performed on intact virions. In total, we identified 31 cellular proteins that were detected in at least two of the three replicates (Table 3). Among these proteins, only 11 proteins were still detected after proteinase K-treatment (Table 3). Manual categorization of the identified proteins according to their previously known molecular function was performed as described for analyses of BoHV-4 [52] and PRV virions [30]. As for these viruses, many proteins involved in cellular signalling, cytoskeleton organization and membrane organization and trafficking were found (Table 3).

**Table 3:** Cellular proteins detected in MuHV-4 virions and comparison to other herpesviruses.

		RelativeeMPAI (%)^a^	pK^c^	*Gammaherpesvirinae*					*Alphaherpesvirinae*		*Beta-* b
				Rhadinoviruses		Maca- b	Lymph- b	Simpl-b		Varicel- b	Cyto- b
Protein description	Acc. N°			KSHV d	BoHV-4 d	AlHV-1 d	EBV d	HSV-1 d		PRV d	HCMV d
*Small GTPases*											
Rab-1A isoform 1	gi|4758988	9.7	**-**				+				
Rab-6A isoform 2	gi|13195674	10.3	**+**					+		+	
Rab GDP diss.inhib.beta-like	gi|354465044	0.9	**+**								+
Immunity-ass.nucleotide 4	gi|13569476	1.2	**-**								
Vesicle transport protein	gi|344256341	2.0	**-**								
ADP-ribosylation factor 2	gi|6671571	8.7	**-**							+	
Ras-related protein Rab-14	gi|16758368	4.3	**+**							+	
*Signalling*											
Casein kinase II subunit beta	gi|7106277	3.1	**-**					+		+	+
Annexin A3	gi|344255510	1.3	**+**								
CD 81 antigen	gi|194135609	10.5	**-**								
S/T-protein Pase PP1-alpha	gi|4506003	5.3	**+**							+	+
Thy-1 membrane glycoprotein	gi|344240805	3.0	**-**							+	
*Cytoskeleton*											
Cofilin-2	gi|6671746	3.6	**-**				+				+
Microtubule-ass prot.RP	gi|7106301	3.1	**-**								
Protein 88B	gi|109463441	0.4	**-**								
Moesin	gi|344240889	1.4	**-**	+			+				
Tetraspanin	gi|685221	1.8	**-**								
*DNA binding*											
Histone H2A type 1	gi|344240017	14.9	**+**								
Histone H4	gi|344240018	3.7	**-**			+					
*Membrane organization and trafficking*											
VAMP 3	gi|6678553	12.3	**+**							+	
** Annexin A2** e	**gi|6996913**	**8.3**	**+**	**+**	**+**	**+**		**+**		**+**	**+**
** Annexin 1**	**gi|235879**	**2.0**	**-**	**+**	**+**	**+**		**+**			**+**
*Metabolism*											
GAPDH	gi|56188	2.2	**+**	+			+	+		+	
F1-ATPase beta subunit	gi|203033	1.3	**-**								
*Ion channel*											
VDAC-1	gi|344238883	2.8	**-**								
*Cell adhesion*											
Protein CLEC16A-like	gi|348584902	0.4	**-**								
*Protein folding and protease*											
PPIase A	gi|351706205	13.0	**+**								
Anionic trypsin-1 precursor	gi|6981420	4.3	**+**								
Polyubiquitin	gi|1050930	4.7	**-**								+
GroEL_like type I chaperonin	gi|51452	0.8	**-**								
*Immunity*											
C4BP	gi|354471099	0.6	**-**								

^a^ Relative eMPAI values were calculated as the eMPAI values calculated for each analysis relatively to the abundance of pORF25 taken as 100%.

^b^ *Beta-*, *Beta-herpesvirinae*; Maca-, Macaviruses; Lymph-, lymphocryptoviruses; Simpl-, simplexviruses; Varicel-, varicelloviruses; Cyto-, cytomegaloviruses.

c pK: Proteinase K treatment. +, proteins detected in the Proteinase K - 1D gel/nanoLC-MS/MS.

d Based on previously published studies.

e Proteins previously identified in MuHV-4 virions [26] are highlighted in bold.

We have chosen to investigate more in depth the role of Annexin A2 in the MuHV-4 cycle for the following reasons: (i) the estimation of protein abundance by emPAI revealed that Annexin A2 is one of the more abundant host proteins detected in MuHV-4 virions; (ii) among the host proteins that were still detected after proteinase K treatment, only Annexin A2 had previously been associated with MuHV-4 virions [26]; (iii) finally, Annexin A2 is the protein that is the most frequently associated with herpesvirus virions (Table 3). Based on these observations, it was therefore plausible that Annexin A2 plays a role in MuHV-4 biology. Interestingly, mouse deficient for Annexin A2 have been generated previously [37]. This was therefore a unique opportunity to investigate more in depth the role of this protein in the MuHV-4 cycle.

**Figure 5:**
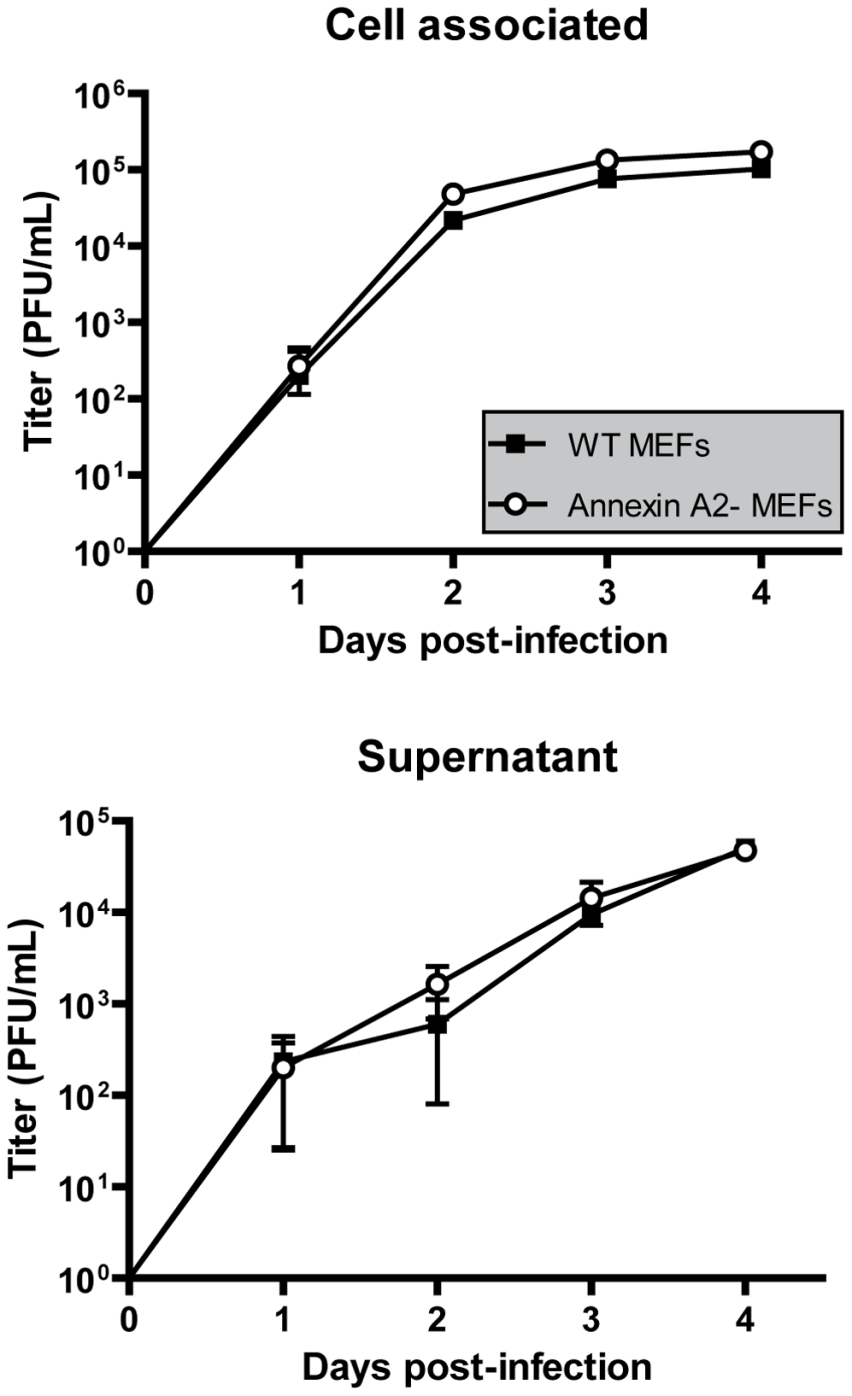
Growth of MuHV-4 on Annexin A2 deficient mouse embryonic fibroblasts. MEF cells from WT and Annexin A2-null mice were infected with BAC+ MuHV-4 virions at a MOI of 0.01 for multi-step assay as described in the Materials and Methods. Supernatants of infected cultures or infected cells were harvested at successive intervals and the amount of infectious virus determined by plaque assay on BHK-21 cells. Plaques were visualized by fluorescent detection of eGFP. The data presented are the average ± SEMs for triplicate measurements. The data were analyzed by 2way ANOVA and Bonferroni posttests. MuHV-4 growths on these two different cell types were not statistically different.

To address this question, we compared the growth of WT MuHV-4 virus on WT or Annexin A2 deficient MEF cells. The results obtained showed that MuHV-4 grew to similar titers in WT and Annexin A2 deficient-MEF cells (Fig. 5). Finally, as Annexin A2 has been proposed to play a role in the entry of different viruses including herpesviruses, we examined MuHV-4 entry into WT and Annexin A2 deficient MEF cells. Briefly, we incubated WT or Annexin A2 deficient MEF cells with a BoHV-4 WT strain expressing eGFP (0.4 PFU/cell) for various times before washing the cells with PBS or with an acidic solution (PBS pH3). The cells were then incubated in complete medium and the numbers of infected cells (eGFP+) were then determined by flow cytometry 18 h later. We did not observe any difference in MuHV-4 entry between the two cell types (data not shown).

## Discussion

The composition of herpesvirus virions has already been addressed by several studies. However the diversity of the purification and mass spectrometry procedures used has led to variable results. While analyses of Herpes-Simplex (HSV-1) and Pseudorabies (PRV) virions identified respectively 44 and 47 viral structural proteins [30], [31], analyses of *rhadinoviruses* gave much more variable results. Thus, 37, 33, 25 and 14 viral structural proteins were revealed in BoHV-4 [36], Rhesus rhadinovirus (RRV) [32], KSHV [25], [34] and MuHV-4 [26] extracellular virions respectively. Moreover, only 10 proteins were common between the different analyses. As mass spectrometry approaches are continuously evolving, we have reassessed the composition of MuHV-4 virions. Our analyses allowed the identification of 31 structural proteins encoded by the MuHV-4 genome which were classified as capsid (8), envelope (9), tegument (13) and unclassified (1) structural proteins. Among these 31 proteins, 27 were also found in at least three of the four rhadinovirus virions characterized to date (BoHV-4, KSHV and RRV) (Table S1).

### Capsid Proteins

Despite considerable sequence divergence, the structure and arrangement of the capsid proteins is remarkably conserved across *Herpesviridae* [9], [53]. Herpesviruses have a T16 icosahedral capsid. The main component of the capsid is the major capsid protein (MCP), encoded by ORF25 in rhadinoviruses. MCP makes up both capsomere structures, the pentons (n = 11) and hexons (n = 150), which contain five and six MCP monomers, respectively. In the capsid, the hexons form the faces and edges while pentons are located at 11 of the vertices of the icosahedron. The last vertice is unique and consists of 12 copies of a portal protein (ORF43 in rhadinoviruses). These portal proteins are arranged as a ring through which viral DNA is encapsidated. The pentons and hexons are bridged together by 320 triplexes which are composed, in rhadinoviruses, of two copies of pORF26 and one copy of pORF62. The capsid is firstly assembled (procapsid) around a morphogenic scaffolding core made of pORF17.5 which undergoes proteolysis by pORF17 during maturation. Finally, a small basic capsid protein (pORF65) is added only after procapsid maturation. Two additional minor capsid proteins, pORF19 and pORF32, associate with capsid triplexes and form the capsid vertex-specific complex (CVSC) which is necessary for viral DNA cleavage and packaging. In this study, we identified all of these proteins (Tables 1 and 2). Analysis of the protein abundances (based on emPAI) conformed to the theoretical values excepted for pORF43, pORF62 and pORF65 which were more abundant than expected. We do not have any explanation for this high relative abundance compared to the other capsid proteins. However, ORF65 encodes the small capsid protein of rhadinoviruses which decorates the hexon configuration of the major capsid protein. On the basis of full occupancy, it should be a very abundant capsid protein. Our results are therefore in accordance with this hypothesis. In contrast, we had shown that pORF65 was not abundant in BoHV-4 virion [36]. This could reveal different roles of this protein in these two species. Interestingly, pORF65 homologues are non-essential in *alpha*- and *beta-herpesvirinae*, but KSHV pORF65 is required for capsid formation [54]. The roles of pORF65 in BoHV-4 and MuHV-4 could therefore be different and will require further investigation.

Similarly to what we observed with BoHV-4, none of the viral terminase components, pORF7, pORF29 and pORF67.5, were detected in our analysis of mature MuHV-4 virions in contrast with KSHV and human cytomegalovirus (HCMV) and with the previous analysis on MuHV-4 virions (Table S2) [26], [34], [35]. This result reinforces therefore the model in which the herpesvirus terminase complexes dissociate from the C-capsid after genome encapsidation. Moreover, the absence of the terminase complex, the high levels of pORF65 and the very low levels of pORF17.5 (homologous to HSV-1 VP22a) (Fig. S1) suggest that our virus preparations contained mostly mature extracellular virions.

### Tegument Proteins

In contrast to the capsid, herpesvirus tegument remains largely undefined. The first step toward the understanding of its organization is therefore the identification of all of its components. Our analysis identified 13 potential viral tegument proteins associated with MuHV-4 virions (proteins encoded by ORFs 11, 21, 23, 33, 36, 38, 45, 52, 55, 63, 64, 75c and 75b). Among these proteins, proteins encoded by ORFs 11, 21, 33, 45, 52, 63, 64 and 75c have all been detected in KSHV tegument [25], [34], [55] while proteins encoded by ORFs 23, 36, 38, 55 and 75b were not. Although being conserved among gammaherpesviruses, the function of ORF23 is unknown and a recent study of MuHV-4 showed that pORF23 was not essential for *in vitro* or *in vivo* growth [56]. Its presence in the MuHV-4 virion is possible based on its similarity with the tegument protein pUL21 of alphaherpesviruses which is involved in egress of capsids from the nucleus [57]. ORF36 encodes a conserved gammaherpesvirus protein kinase [58], [59] which has also been detected in BoHV-4 [36] and RRV [32]. Proteins encoded by ORF38 and ORF55 are fatty acylated proteins conserved throughout the herpesvirus family. They have been detected in the tegument of many herpesviruses and are involved in virion envelopment and/or egress [60], [61]. Finally, pORF75b has been described as a virion component [62]. All these proteins, except pORF55, were detected after proteinase K treatment of virions (Table 1) and should therefore be considered as real tegument components. pORF55 was only detected by two peptides and its absence in proteinase K treated virions likely reflects its low abundance in viral particles.

In contrast to these proteins, our analyses did not detect proteins encoded by ORFs 6, 20, 35, 42, 48, 50, 67 and 75a which had previously been described as potential tegument proteins in KSHV [55]. In particular, pORF20 [26], pORF42 [63] and pORF75a [62] had previously been associated with MuHV-4 virions. Our results suggest that these proteins are absent or extremely rare in MuHV-4 virions. The absence of pORF75a was quite surprising as its two homologues, pORF75c and pORF75b, were among the most abundant proteins of MuHV-4 virions (on the basis of the emPAI values). This therefore suggests that these proteins have diverged to acquire new functions as proposed by Gaspar *et al.* who observed that these proteins do not substitute functionally.

As observed in our analysis of BoHV-4 virions [36], the most abundant MuHV-4 virion protein is encoded by ORF52 (Table 1). ORF52 is conserved in *gammaherpesvirinae* and encodes a small protein of ∼20 kDa. In MuHV-4, ORF52 is essential for tegumentation and secondary envelopment [26], [63]–[65]. pORF52 seems to function as a dimer and the N-terminal α-helix is likely involved in interactions with other virion components [66] such as pORF33, pORF75, gM and gN in KSHV [67] and pORF42 in MuHV-4 [63]. Even if pORF52 is a very abundant component of MuHV-4 virion [64], its abundance in MuHV-4 could be overestimated by the emPAI value. Indeed, emPAI calculation integrates an intrinsic property of a protein which is the number of peptides that will be generated through trypsin digestion. However, the nature of the tryptic peptides and in particular their capacity to get ionized in the mass spectrometer ion source is not taken into account. pORF52 is a highly basic protein (pI around 10) and its tryptic peptides could be more efficiently ionized. These peptides could therefore appear as more abundant during mass spectrometry analysis. EmPAI has therefore to be considered as an estimation of the abundance. Within a sample, the relative proportions have to be mainly used to classify proteins based on their abundance.

Based on emPAI analysis MuHV-4 pORF52 appears even tenfold more abundant in virion than BoHV-4 pORF52. However, mass spectrometry relies on *on the flight* selection by the instrument of most abundant peptides to be fragmented and identified. This selection can thus be biased by numerous experimental condition factors such as the efficiency of protein extraction or the method used for their separation. Therefore, comparison of quantitative data concerning different proteins in different biological matrices (BoHV-4 pORF52 and MuHV-4 pORF52) has to be considered cautiously and can be subject to numerous experimental bias. The main conclusion that can therefore be drawn from the analysis of pORF52 abundance in MuHV-4 and BoHV-4 [36] virion analyses is that pORF52 is the most abundant protein in virions of both species.

Finally, among these potential tegument proteins, 11 were copurified with capsids (Table 2). The two proteins that were not detected after removal of envelopes are pORF38 and pORF55. This could be linked to their low abundance as only a few peptides corresponding to these proteins were detected in entire virion samples. However, pORF38 was also not detected in a similar BoHV-4 capsid-tegument preparation [36]. This could be explained by the association of pORF38 with some envelope glycoproteins, as observed for its HSV-1 homologue pUL11 [68].

### Envelope Proteins

The approach used in this study allowed the detection of 9 MuHV-4 envelope proteins encoded by ORFs 4, 8, 22, 27, 28, 39, 47, 51 and 58. These proteins were categorized as such based on the extensive literature about them, on the presence of at least one predicted transmembrane domain (excepted for gL encoded by ORF47) and on their disappearance after removal of virus envelope (Tables 1 and 2). Comparison of the relative abundance of these proteins suggests that pORF4 (gp70), pORF8 (gB), pORF22 (gH), pORF27 (gp48), pORF28 and pORF39 (gM) have comparable abundance as their relative emPAI values (in comparison with pORF25, the major capsid protein) range from 19 to 46%. In contrast, the relative abundance of pORF47 (gL) was lower. As observed in other viruses, MuHV-4 gL associates with gH to form a heterodimer that plays a central role in virus binding [18] and in membrane fusion [69]. Our previous results suggested that gH exists in two distinct forms at the surface of MuHV-4 virions, either in association or not with gL [69]. This fact could explain the difference of abundance between the two proteins that we observed in this study. The relative abundance of pORF51 (gp150) and pORF58 was also very low. In the case of gp150, this apparent low abundance could be due to the high degree of glycosylation of this protein as proposed for the homologous BoHV-4 gp180 [36]. Concerning pORF58, this could be linked to the low penetration of the protein in the electrophoresis gel (Fig. 4) or to the formation of protein aggregates as described above. Most of the detected envelope proteins appeared to be glycosylated (Fig. 4).

Similar to what we observed with BoHV-4 [36], we did not detect any peptide corresponding to gN which is encoded by ORF53 (Table S2). gN and gM form a complex in herpesviruses [39], [70] and gN is needed for the proper processing of gM. gN could dissociate from gM in mature virions as suggested for HCMV [35]. However, it is not the case here as gN was readily detected by western blotting on MuHV-4 virions even after proteinase K treatment (Fig. 2). The absence of gN in our analysis is therefore likely due to a detection failure by our mass spectrometry approach. Similarly to what was observed for KSHV and RRV, the viral G-protein coupled receptor encoded by ORF74 has not been found in MuHV-4 virions.

Finally, the protein encoded by the M3 gene was found in all our analyses of complete virions (n>10) even after proteinase K treatment but not after deglycosylation (Table 1). M3 encodes a MuHV-4 specific secreted 44 kDa which is highly expressed during lytic infection [71]. This protein has a broad chemokine-binding activity [72], [73] and could prevent the migration of CD8^+^ T cells into sites where immunogenic latency antigens are expressed. In accord with this suggestion, M3 disruption resulted in reduced amplification of latently infected B cells [74], [75]. However, the effect on lytic replication was less clear [75], [76]. Our results suggest that M3 could be associated with the surface of MuHV-4 virions. Interestingly, glycoproteins G of alphaherpesviruses are chemokine-binding proteins that are present on the viral particle and on the plasma membrane of infected cells [77], [78]. Although gGs are virulence factors [79], the mechanisms beneath this phenotype remain unclear. Some reports highlighted a role in virus entry [80], [81]. MuHV-4 M3 disruption did not lead to an apparent binding deficit in the cell-types that have been tested so far. In the future, binding of M3-deficient virions to a range of cell types will have to be tested.

### Host Proteins Associated with MuHV-4 Extracellular Virions

Host proteins are frequently associated with the structure of virions from various families [82], [83]. Despite some exceptions [23], the functions of these proteins in virus biology are poorly characterized. Host proteins have also been found in most of the analyses of herpesvirus virion composition [24]–[36]. In our analysis of MuHV-4 virions, we found 31 cellular proteins that were detected in at least two of the three replicates (Table 3). Only 11 of these proteins were still detected after proteinase K-treatment (Table 3) and their relative abundances were quite low. Our results suggest therefore that cellular proteins are minor components of MuHV-4 virions. However, several of these proteins are small GTPases (Rab-1A, Rab-6A, Rab-14) or proteins linked to membrane organization and trafficking (VAMP3, Annexin A2) that could reveal the egress pathway used by MuHV-4.

Interestingly, Annexin A2 was also proposed as a constituent of several other herpesviruses (including KSHV, BoHV-4, AlHV-1, HSV-1, PRV and HCMV) as well as influenza [84], HIV-1 [85], papillomavirus [86] and Hepatitis C [87]. Annexin A2 is a 36 kDa Ca^2+^-regulated phospholipid binding protein which exists both free in the cytoplasm and associated with plasma membrane surfaces. Annexins can function as organizers of membrane domains and membrane recruitment platforms for proteins with which they interact [88]. The role of Annexin A2 in herpesvirus infection has mainly been studied with HCMV. Thus, Annexin A2 has been shown to associate with HCMV virions [89], [90] through specific interaction with gB [91], [92]. Although this association is well established, its functional importance is still subject to controversies. Some studies showed that virion incorporated Annexin A2 could accelerate fusion of membranes during entry [93] in accordance with the observation that some Annexin A2 specific antibodies could inhibit HCMV infection [94]. In contrast, other studies showed that Annexin A2 has no effect on HCMV entry into fibroblasts [95]. Our results similarly showed that despite being incorporated in MuHV-4 virions, Annexin A2 does not influence growth of this virus in mouse embryonic fibroblasts (Fig. 5). In the future, these results will have to be confirmed in other cell types as the effect of Annexin A2 on MuHV-4 biology could be cell-specific as recently observed for HIV-1 [96].

**Figure 6:**
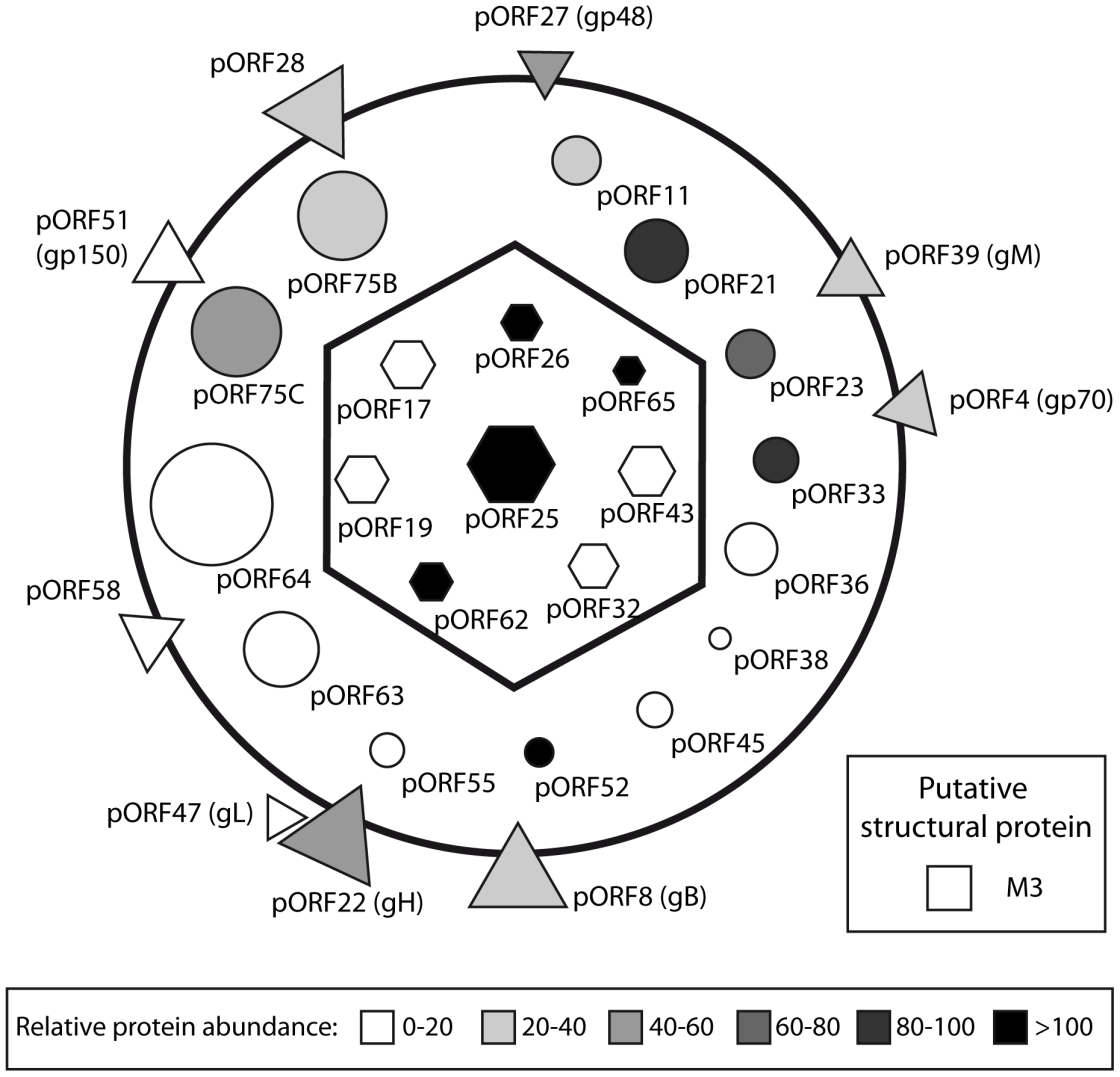
Schematic representation of the protein composition of mature extracellular MuHV-4 virions. Capsid proteins are represented as hexagons, tegument proteins as circles, envelope proteins as triangles. As the location of the M3 protein is unknown, it is represented as a putative structural protein (square). The predicted protein mass is directly proportional to their surface. The mean relative abundance (emPAI) determined by the different analyses of complete virions is indicated in color intensity (see scale).

In summary, we extended our comprehension of the composition of MuHV-4 virions in particular and of rhadinoviruses virions in general. We identified 31 viral proteins as constituents of MuHV-4 virions. Among these proteins, 30 were resistant to proteinase-K treatment. Based on the literature, it appears that this composition is very similar to those of BoHV-4 [36] and of RRV virions [32]. This therefore allowed us to draw a schematic MuHV-4 particle (Fig. 6) which is likely representative of the general architecture of rhadinoviruses. Moreover, we identified 31 cellular proteins as potential structural components of MuHV-4 virions. However these proteins were not abundant and one of them, Annexin A2, which has been detected in many herpesviruses, was non-essential to viral growth *in vitro*. In the future, these results could improve our knowledge about different steps of the biology of gammaherpesviruses.

## Supporting Information

Figure S1 **Analysis of peptides identified in the different expression products of MuHV-4 ORF17-17.5 locus.** The predicted product of expression of ORF17 and ORF17.5 and their respective cleavage products are represented by rectangles. Black bars indicate the tryptic fragments identified by mass spectrometry. Open triangles indicate protease cleavage sites.
(TIF)

Figure S2 **Deglycosylation of MuHV-4 virions.** Purified MuHV-4 virions were either left untreated (/) or deglycosylated (N−/O-) as indicated in the Materials and Methods. The samples were then immunoblotted with mAbs 3F7 (anti-gN), 58-16D2 (anti-gp70), MG-4D11 (anti-gB C-terminal end), MG-2C10 (anti-gB N-terminal end) or with rabbit polyserum raised against MuHV-4 virions.
(TIF)

Figure S3 **Prediction of N-glycosylation sites for the different MuHV-4 protein sequences detected by our approach using the NetNglyc 1.0 and NetOglyc 3.1 algorithms.** The red lines indicate significative threshold. Vertical blue lines indicate potential glycosylation sites.
(TIF)

Table S1 **Comparison of MuHV-4 proteins identified in virions with other herpesviruses.**
(PDF)

Table S2 **Comparison of MuHV-4 proteins non-identified in virions with observations in other herpesviruses.**
(PDF)
