# Supplementary figures and images for: Proteomic Characterization of Murid Herpesvirus 4 Extracellular Virions
Source: PLoS One. 2013 Dec 30;8(12):e83842. doi: 10.1371/journal.pone.0083842 (PMC3875534; doi:10.1371/journal.pone.0083842)

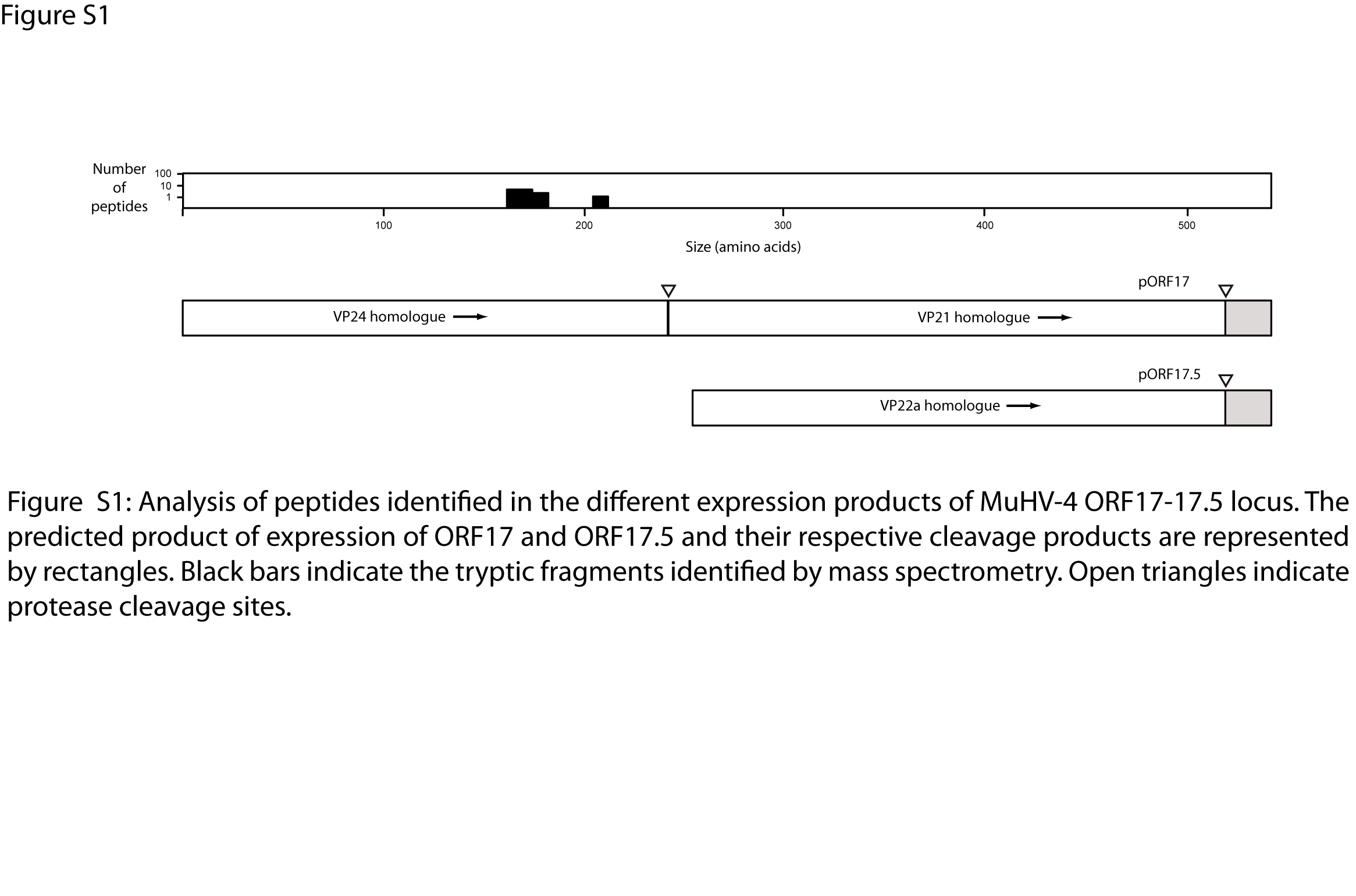

Supplement: Figure S1 — Analysis of peptides identified in the different expression products of MuHV-4 ORF17-17.5 locus. The predicted product of expression of ORF17 and ORF17.5 and their respective cleavage products are represented by rectangles. Black bars indicate the tryptic fragments identified by mass spectrometry. Open triangles indicate protease cleavage sites. (TIF) [file pone.0083842.s001.tif]

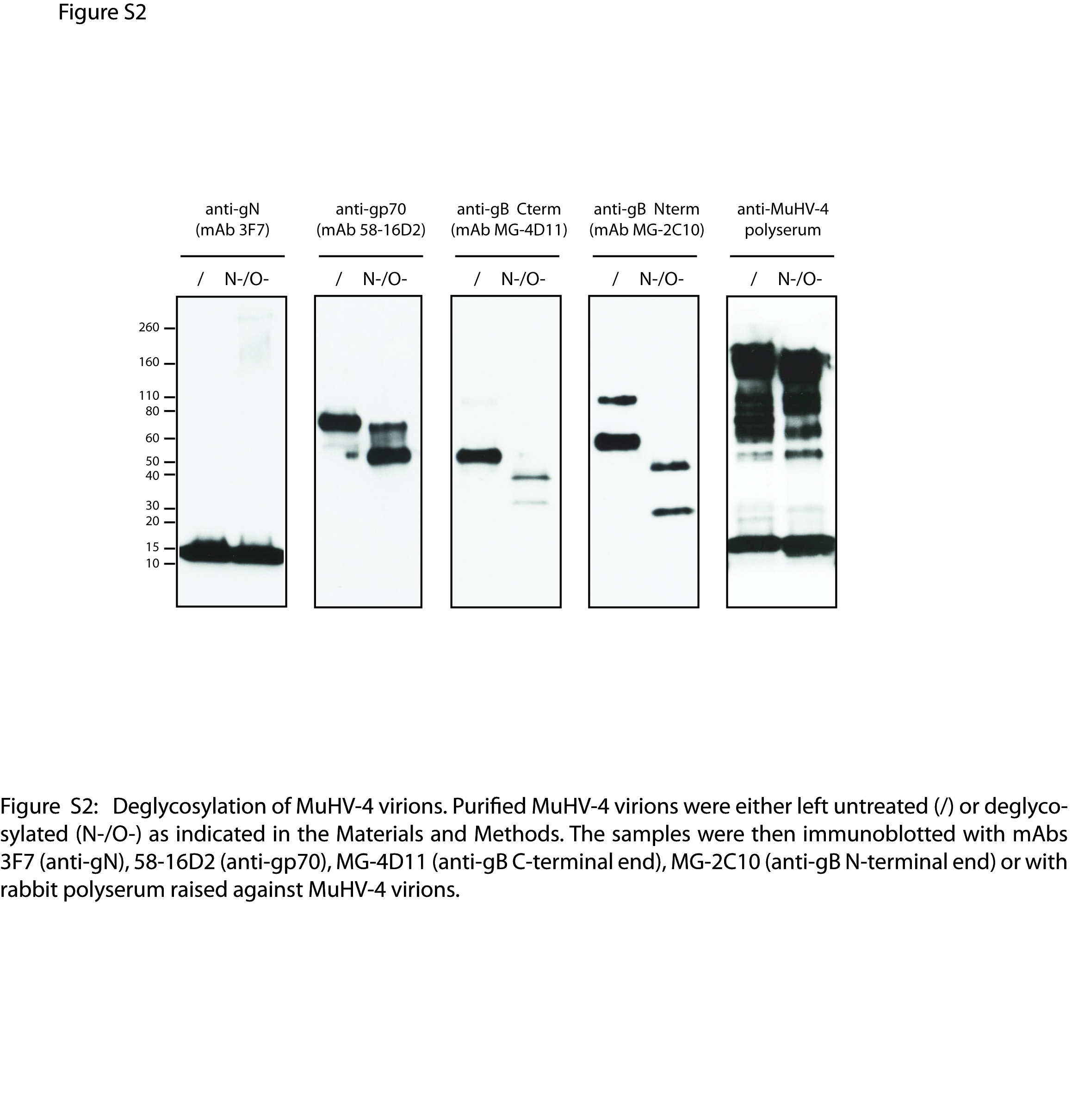

Supplement: Figure S2 — Deglycosylation of MuHV-4 virions. Purified MuHV-4 virions were either left untreated (/) or deglycosylated (N−/O-) as indicated in the Materials and Methods. The samples were then immunoblotted with mAbs 3F7 (anti-gN), 58-16D2 (anti-gp70), MG-4D11 (anti-gB C-terminal end), MG-2C10 (anti-gB N-terminal end) or with rabbit polyserum raised against MuHV-4 virions. (TIF) [file pone.0083842.s002.tif]

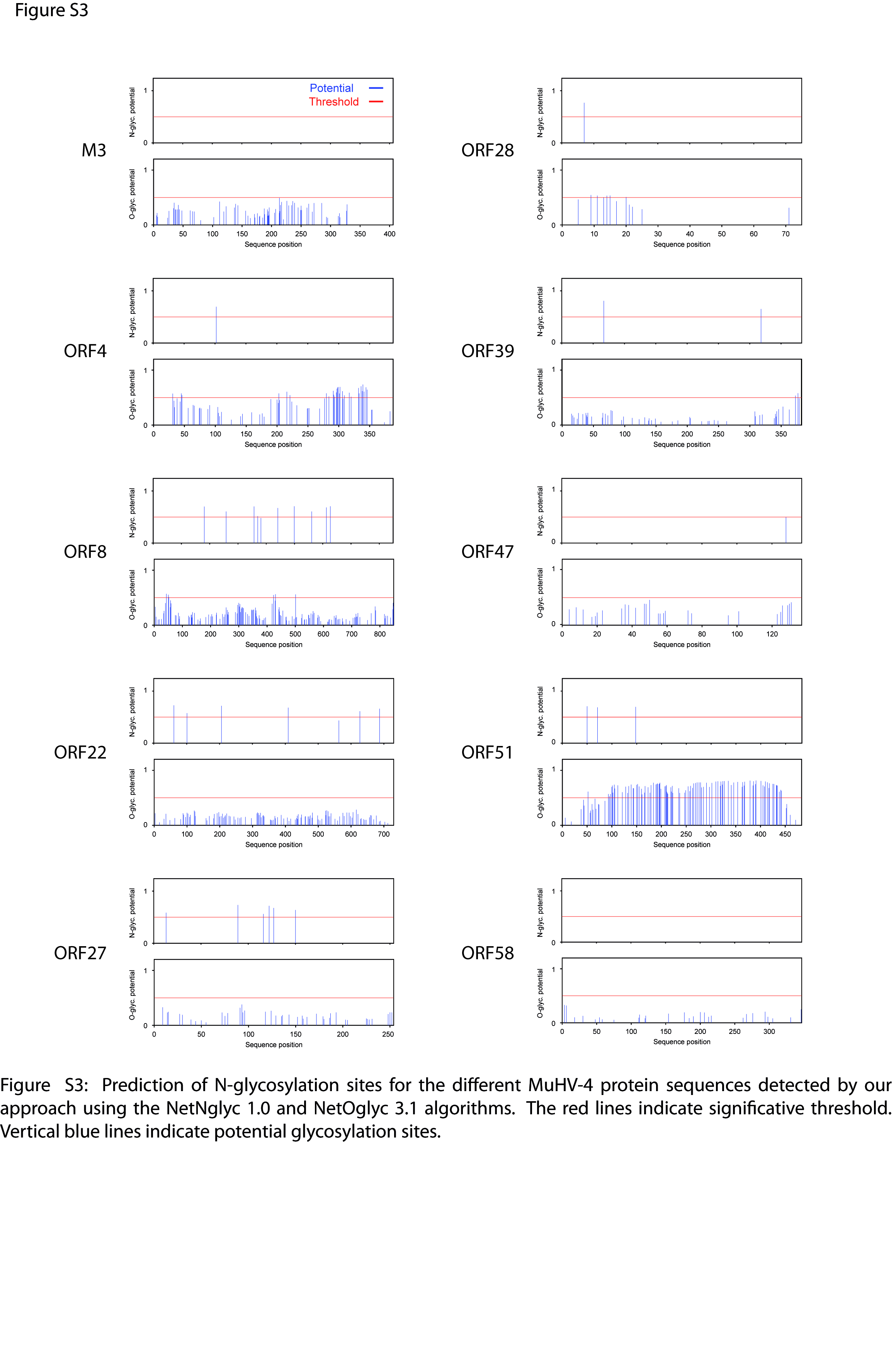

Supplement: Figure S3 — Prediction of N-glycosylation sites for the different MuHV-4 protein sequences detected by our approach using the NetNglyc 1.0 and NetOglyc 3.1 algorithms. The red lines indicate significative threshold. Vertical blue lines indicate potential glycosylation sites. (TIF) [file pone.0083842.s003.tif]
