# Supplementary material for: Proteomic Characterization of Murid Herpesvirus 4 Extracellular Virions
Source: PLoS One. 2013 Dec 30;8(12):e83842. doi: 10.1371/journal.pone.0083842 (PMC3875534; doi:10.1371/journal.pone.0083842)
Supplement: Table S1 — Comparison of MuHV-4 proteins identified in virions with other herpesviruses. (PDF) [file pone.0083842.s004.pdf]

TABLE S1 Comparison of MuHV-4 proteins identified in virions with other herpesviruses[a]

			Gamma-herpesvirinae					*Alpha-herpesvirinae*		*Beta-*[b]
			Rhadinoviruses			Maca-[b]	Lymph-[b]	Simplex-[b]	Varicel-[b]	Cyto-[b]
**ORF**	**Protein description**	**pK**[c]	**KSHV**[d]	**BoHV-4**[d]	**RRV**[d]	**AlHV-1**[d]	**EBV**[d]	**HSV-1**[d]	**PRV**[d]	**HCMV**[d]
Capsids										
ORF17	minor scaffold protein (protease)	+	ORF17	ORF17	ORF17	ORF17	BVRF2	UL26	UL26	UL80
ORF17.5	major scaffold protein	+	ORF17.5	ORF17.5	ORF17.5	ORF17.5	BdRF1	UL26.5	UL26.5	UL80.5
ORF19	capsid vertex-specific complex protein	+	ORF19	ORF19	ORF19	ORF19	BVRF1	UL25	UL25	UL77
ORF25[e]	major capsid protein	+	ORF25	ORF25	ORF25	ORF25	BcLF1	UL19	UL19	UL86
ORF26	triplex component	+	ORF26	ORF26	ORF26	ORF26	BDLF1	UL18	UL18	UL85
ORF32	capsid vertex-specific complex protein	+	ORF32	ORF32	ORF32	ORF32	BGLF1	UL17	UL17	UL93
ORF43	capsid portal protein	+	ORF43	ORF43	ORF43	ORF43	BBRF1	UL6	UL6	UL104
ORF62	triplex component	+	ORF62	ORF62	ORF62	ORF62	BORF1	UL38	UL38	UL46
ORF65	small capsomer interacting protein	+	ORF65	ORF65	ORF65	ORF65	BFRF3	UL35	UL35	UL48.5
Envelope										
ORF4	Glycoprotein gp70	+	ORF4	n/e	ORF4	n/e	n/e	n/e	n/e	n/e
ORF8	glycoprotein B	+	ORF8	ORF8	ORF8	ORF8	BALF4	UL27	UL27	UL55
ORF22	glycoprotein H	+	ORF22	ORF22	ORF22	ORF22	BXLF2	UL22	UL22	UL75
ORF27	Glycoprotein gp48	+	ORF27	n/e	ORF27	ORF27	BDLF2	n/e	n/e	n/e
ORF28	potential glycoprotein	+	ORF28	Bo9	ORF28	n/e	BDLF3	n/e	n/e	n/e
ORF39	glycoprotein M	+	ORF39	ORF39	ORF39	ORF39	BBRF3	UL10	UL10	UL100
ORF47	glycoprotein L	+	ORF47	ORF47	ORF47	ORF47	BKRF2	UL1	UL1	UL115
ORF51	glycoprotein gp150	+	K8.1	Bo10	R8.1	A8	BLLF1	n/e	n/e	n/e
ORF58	glycoprotein	+	ORF58	ORF58	ORF58	ORF58	BMRF2	n/e	n/e	n/e
Tegument and unknown										
M3	Chemokine-binding protein	+	n/e	n/e	n/e	n/e	n/e	n/e	n/e	n/e
ORF11	Potential tegument protein	+	ORF11	n/e	ORF11	ORF11	BILF4	n/e	n/e	n/e
ORF21	thymidine kinase, potential tegument protein	+	ORF21	ORF21	ORF21	ORF21	BXLF1	UL23	UL23	n/e
ORF23	Egress protein, tegument	+	ORF23	ORF23	ORF23	ORF23	BTRF1	UL21	UL21	UL88
ORF33	Teg. myristoylated protein binding protein	+	ORF33	ORF33	ORF33	ORF33	BGLF2	UL16	UL16	UL94
ORF36	kinase	+	ORF36	ORF36	ORF36	ORF36	BGLF4	UL13	UL13	UL97
ORF38	tegument myristoylated protein	+	ORF38	ORF38	ORF38	ORF38	BBLF1	UL11	UL11	UL99
ORF45	IRF-7 binding prot., pot. tegument protein	+	ORF45	ORF45	ORF45	ORF45	BKRF4	n/e	n/e	n/e
ORF52	tegument protein	+	ORF52	ORF52	ORF52	ORF52	BLRF2	n/e	n/e	n/e
ORF55	tegument palmitoylated protein	-	ORF55	ORF55	ORF55	ORF55	BSRF1	UL51	UL51	UL71
ORF63	large tegument protein binding protein	+	ORF63	ORF63	ORF63	ORF63	BOLF1	UL37	UL37	UL47
ORF64	tegument protein	+	ORF64	ORF64	ORF64	ORF64	BPLF1	UL36	UL36	UL48
ORF75C	tegument protein/v-FGAM-synthetase	+	ORF75	ORF75	ORF75	ORF75	BNRF1	n/e	n/e	n/e
ORF75B	tegument protein/v-FGAM-synthetase	+	n/e	n/e	n/e	n/e	n/e	n/e	n/e	n/e

[a] Proteins identified in other virions are higlighted in black. Proteins highlighted in grey have been detected in very low abundance.

[b] *Beta-*, *Beta-herpesvirinae* ; Maca-, Macaviruses ; Lymph-, lymphocryptoviruses ; Simplex, simplexviruses ; Varicel-, varicelloviruses ; Cyto-, cytomegaloviruses.

[c] Proteinase K treatment. +, proteins detected in the proteinase K – 1D gel/nanoLC-MS/MS analysis.

[d] based on previously published studies [24-36].

[e] Proteins previously identified in MuHV-4 virions [26] are highlighted in light grey.
