# Supplementary material for: Proteomic Characterization of Murid Herpesvirus 4 Extracellular Virions
Source: PLoS One. 2013 Dec 30;8(12):e83842. doi: 10.1371/journal.pone.0083842 (PMC3875534; doi:10.1371/journal.pone.0083842)
Supplement: Table S2 — Comparison of MuHV-4 proteins non-identified in virions with observations in other herpesviruses. (PDF) [file pone.0083842.s005.pdf]

TABLE S2 Comparison of MuHV-4 proteins non-identified in virions with observations in other herpesviruses[a]

		Gammaherpesvirinae					*Alphaherpesvirinae*		*Beta-*[b]
		Rhadinoviruses			*Maca-*[b]	*Lymph-*[b]	Simplex-[b]	Varicel-[b]	Cyto-[b]
ORF	Protein description	KSHV[c]	BoHV-4[c]	RRV[c]	AlHV-1[c]	EBV[c]	HSV-1[c]	PRV[c]	HCMV[c]
ORF6	Single-stranded DNA-binding protein MDBP	ORF6	ORF6	ORF6	ORF6	BALF2	UL29	UL29	UL57
ORF7	Terminase	ORF7	ORF7	ORF7	ORF7	BALF3	UL28	UL28	UL56
K3	Homolog of KSHV K5 (E3 ubiquitin ligase)	K5	Bo5	n/e	n/e	n/e	n/e	n/e	n/e
ORF9	DNA polymerase	ORF9	ORF9	ORF9	ORF9	BALF5	UL30	UL30	UL54
ORF10	dUTPase related protein	ORF10	ORF10	ORF10	ORF10	n/e	n/e	n/e	n/e
ORF20[d]	Inactivator of the Cdc2–cyclin B complex	ORF20	ORF20	ORF20	ORF20	BXRF1	UL24	UL24	UL76
ORF24	Regulator of late genes expression	ORF24	ORF24	ORF24	ORF24	BcRF1	n/e	n/e	UL87
ORF29	Terminase	ORF29	ORF29	ORF29	ORF29	BGRF1	UL15	UL15	UL89
ORF35	Potential tegument protein (UL14 homologue)	ORF35	ORF35	ORF35	ORF35	BGLF3.5	UL14	UL14	UL95
ORF42	Potential tegument protein (UL7 homologue)	ORF42	ORF42	ORF42	ORF42	BBRF2	UL7	UL7	UL103
ORF46	Uracyl DNA glycosylase	ORF46	ORF46	ORF46	ORF46	BKRF3	UL2	UL2	UL114
ORF48	Potential tegument protein	ORF48	ORF48	ORF48	ORF48	BRRF2	n/e	n/e	n/e
ORF49	RTA cooperating protein	ORF49	ORF49	ORF49	ORF49	BRRF1	n/e	n/e	n/e
ORF50	Replication and transcription activator protein	ORF50	ORF50	ORF50	ORF50	BRLF1	n/e	n/e	n/e
ORF53	Glycoprotein N	ORF53	ORF53	ORF53	ORF53	BLRF1	UL49.5	UL49.5	UL73
ORF54	dUTPase	ORF54	ORF54	ORF54	ORF54	BLLF3	UL50	UL50	UL72
ORF57	Posttranscriptional regulatory protein	ORF57	ORF57	ORF57	ORF57	BMLF1	UL54	UL54	UL69
ORF59	DNA replication protein (Processivity factor)	ORF59	ORF59	ORF59	ORF59	BMRF1	UL42	UL42	UL44
ORF60	Ribonucleotide reductase small subunit	ORF60	ORF60	ORF60	ORF60	BaRF1	UL40	UL40	n/e
ORF61	Ribonucleotide reductase large subunit	ORF61	ORF61	ORF61	ORF61	BORF2	UL39	UL39	UL45
ORF67	Potential tegument protein	ORF67	ORF67	ORF67	ORF67	BFRF1	UL34	UL34	UL50
ORF67.5	Terminase	ORF67.5	ORF67.5	ORF67.5	ORF67.5	BBRF1	UL33	UL33	UL51
ORF68	DNA packaging protein	ORF68	ORF68	ORF68	ORF68	BFLF1	UL32	UL32	UL52

[a] Proteins identified in other virions are higlighted in black. Proteins highlighted in grey have been detected in very low abundance.

[b] *Beta-*, *Beta-herpesvirinae* ; Maca-, Macaviruses ; Lymph-, lymphocryptoviruses ; Simplex, simplexviruses ; Varicel-, varicelloviruses ; Cyto-, cytomegaloviruses.

[c] based on previously published studies [24-36].

[d] Proteins previously identified in MuHV-4 virions [26] are highlighted in light grey.
